# CircRNA has_circ_0001806 promotes hepatocellular carcinoma progression via the miR-193a-5p/MMP16 pathway

**DOI:** 10.1590/1414-431X2021e11459

**Published:** 2021-10-29

**Authors:** Hongmu Zhou, Ying Chen

**Affiliations:** 1 Department of Geriatrics, General Hospital of The Yangtze River Shipping, Wuhan Brain Hospital, Wuhan, Hubei, China; 2 Department of Gastroenterology, Affiliated Hospital of Jianghan University, Wuhan Sixth Hospital, Wuhan, Hubei, China

**Keywords:** Circ_0001806, Hepatocellular carcinoma, MMP16, miR-193a-5p

## Abstract

Reportedly, circular RNAs (circRNAs) are crucial regulators in cancer progression. Nonetheless, the molecular mechanism of circRNAs in hepatocellular carcinoma (HCC) has not been fully clarified. Gene expression omnibus (GEO) database was employed to screen out the differentially expressed circRNAs in HCC. qRT-PCR and western blot were executed to detect circ_0001806 expression, miR-193a-5p expression, and MMP16 mRNA and protein expressions in HCC. The effect of circ_0001806 on HCC was analyzed by the CCK-8 method and Transwell experiment. RIP assay, pull-down experiment, and dual-luciferase reporter gene experiment were applied to validate the targeting relationships among circ_0001806, miR-193a-5p, and MMP16. Circ_0001806 was up-modulated in HCC tissues and cell lines. Knockdown of circ_0001806 impeded the multiplication, migration, and invasion of HCC cells. Circ_0001806 could up-regulate MMP16 expression through repressing miR-193a-5p, thereby facilitating the malignant biological behaviors of HCC. Circ_0001806 promoted HCC progression by regulating miR-193a-5p/MMP16 axis.

## Introduction

Liver cancer has become the sixth most frequently diagnosed cancer and the fourth leading cause of cancer-related death in the world (1). Hepatocellular carcinoma (HCC) accounts for 75-85% of cases of primary liver cancer. Since early HCC is usually asymptomatic, HCC patients are usually at an advanced stage when diagnosed and have missed the opportunity for curative treatment (liver resection or liver transplantation) (2,3). The high recurrence rate after radical resection and chemoresistance of HCC are major challenges in the treatment of HCC (3,4). To improve the diagnosis, prevention, and treatment of HCC, in-depth research into the molecular mechanism of this disease is therefore of great importance.

Circular RNAs (circRNAs) are RNA transcripts with a covalently closed circular structure that lack a 5′ cap and 3′ tail. The sequence of circRNAs is conserved, the structure of circRNAs is stable, and the expression of circRNA is tissue-specific (5). These characteristics endow circRNAs with many potential functions, such as acting as a microRNA (miRNA) sponge, or combining with proteins to form RNA-protein complexes to modulate the function of proteins (6). Accumulating research has demonstrated that circRNAs are related to the pathogenesis of tumors (7-10). Importantly, circRNA can function as competitive endogenous RNA (ceRNA) to regulate the expression level of miRNAs and target genes (6). For instance, circSLC8A1 is reported to directly interact with miR-130b or miR-494, acting as an miRNA sponge to modulate PTEN expression and downstream signaling pathways, thereby restraining bladder cancer progression (11). Nevertheless, the role and mechanism of circRNA in HCC have not been fully clarified.

In this work, we identified a circRNA, has_circ_0001806, that was significantly up-modulated in HCC tissues based on the circRNA microarray data. Further research revealed that circ_0001806 was highly expressed in HCC tissues and cell lines. Circ_0001806 knockdown impeded the growth, migration, and invasion of HCC cells. Additionally, circ_0001806 worked as a ceRNA for miR-193a-5p to up-modulate matrix metallopeptidase 16 (MMP16).

## Material and Methods

### Tissues specimens

This study was approved by the Ethics Committee of the Affiliated Hospital of Jianghan University. Tissue specimens were collected from patients who were diagnosed with HCC between May 2014 and May 2019 at the Affiliated Hospital of Jianghan University, and stored in liquid nitrogen at -196°C. Written informed consent was signed by each patient. Two HCC microarray datasets (GSE94508 and GSE97332) were downloaded from the Gene Expression Omnibus (GEO) database.

### Cell lines, cell culture, and transfection

Normal hepatic cell line (L02) and HCC cell lines (Huh-7, HepG2, SMMC-7721, Bel-7402, and HL-7702) were procured from the Cell Bank of the Chinese Academy of Sciences (China). Cells were cultured in Dulbecco’s modified Eagle’s medium (DMEM) (Thermo Fisher, USA) supplemented with 10% fetal bovine serum (FBS) (Gibco, USA), 100 μg/mL streptomycin and 100 U/mL penicillin (Invitrogen, USA) at 37°C in 5% CO_2._ In a 6-well plate, the HCC cells were planted at 3×10^5^ cells/well. The cells were transfected with the negative control of siRNA (si-NC, 5′-CCAGUUUACCUAACGCAAUTT-3′); siRNA targeting circ_0001806 (si-circ_0001806#1: 5′-GTCTCCCAGTGCTCCAGACAA-3′; si-circ_0001806#2: 5′-TCCCAGTGCTCCAGACAATGA-3′; si-circ_0001806#3: 5′-TCTCCCAGTGCTCCAGACAAT-3′); negative control of miR-193a-5p mimic (mimic-NC, 5′-UCACAACCUCCUAGAAAGAGUAGA-3′); miR-193a-5p mimic (miR-193a-5p, 5′- UGGGUCUUUGCGGGCGAGAUGA-3′); miR-193a-5p inhibitor (anti-miR-193a-5p, 5′-UCAUCUCGCCCGCAAAGACCCA-3′); and negative control of miR-193a-5p inhibitor (anti-miR-control, 5′-CAGUACUUUUGUGUAGUACAA-3′) using Lipofectamine^®^ 2000 (Invitrogen), when the cell confluence reached 90%. The aforementioned oligonucleotides were available from GenePharma Co., Ltd. (China).

### Quantitative real-time polymerase chain reaction (qRT-PCR)

Total RNA in tissues and cells was isolated by TRIzol reagent (Invitrogen), and the purity and concentration of RNA were measured by NanoDrop 2000 (Thermo Fisher Scientific Inc.). With the ReverTra Ace qPCR RT Package (Toyobo, Japan), RNA was transcribed into cDNA. With cDNA as template, qRT-PCR was conducted with THUNDERBIRD SYBR qPCR Mix (Toyobo). The gene expression was normalized by GAPDH/U6 expression using the 2^−ΔΔCt^ method. The primers designed for this research are displayed in Table 1. To validate the circular structure of circ_0001806, total RNA (5 μg) was incubated at 37°C for 15 min with 3 U/μg of RNase R (Epicentre Biotechnologies, China), and subsequently qRT-PCR was performed, with GAPDH as the control of linear RNA.

**Table 1:** Primer sequences used in this study.

Gene	Sequence
circ_0001806	Forward: 5-GTGATCTGAAAGGGCCAGAG-3
	Reverse: 5-TCCACATCACCCTTCACCTT-3
MMP16	Forward: 5-TAGAGCGTGCAGATAATGACAAGGA-3
	Reverse: 5-TGAACTGCTAGCCTCTGGATTTGA-3
miR-193a-5p	Forward: 5-TATATGGGTCTTTGCGGGCG-3
	Reverse: 5-GTGCAGGGTCCGAGGT-3
GAPDH	Forward: 5-GGAGCGAGATCCCTCCAAAAT-3
	Reverse: 5-GGCTGTTGTCATACTTCTCATGG-3
U6	Forward: 5-ATTGGAACGATACAGAGAAGATT-3
	Reverse: 5-GGAACGCTTCACGAATTTG-3

### Cell counting kit-8 (CCK-8) experiment

Huh-7 and HepG2 cells were seeded onto 96-well plates (1.0×10^3^ cells/well) and cultured for 0, 24, 48, and 72 h. Then, 10 μL of CCK-8 solution (Dojindo, Japan) was added to each well. Cells were incubated for 1 h, and the absorbance (at 450 nm wavelength) of each well was examined by a microplate reader (Bio-Rad, USA).

### Migration and invasion experiments

Approximately 1×10^4^ transfected cells were suspended in 200 μL of serum-free medium and positioned in the upper compartment of each Transwell chamber (8-μm pore size, USA). A medium containing 10% FBS was added to the lower compartment as the chemoattractant. Cells were incubated at 37°C with 5% CO_2_ for 48 h for the invasion experiment and 24 h for the migration experiment. Then, cells in the upper compartment were removed with cotton swabs and cells on the lower surface of the filter were fixed with methanol and stained with 0.1% crystal violet. Within 3 random areas, the number of the cells was counted under a microscope. Matrigel (BD Biosciences, USA) was used to cover the filter in the invasion assay, but was not used in the migration assay.

### Dual-luciferase reporter experiment

HEK293T cells were planted onto 96-well plates (1×10^4^ cells/well), cultured at 37°C for 12 h, and then co-transfected with wild type (WT) or mutant type (MUT) luciferase reporter vectors and miR-193a-5p mimics or mimic-NC using Lipofectamine^®^ 2000 (Invitrogen). After 48 h, the Firefly and Renilla luciferase activities were tested by a Dual-Luciferase Reporter Assay kit (Promega, USA).

### RNA immunoprecipitation (RIP) experiment

The RIP experiment was executed with an EZ-Magna RIP kit (EMD Millipore, USA). RIP lysis buffer plus cocktail (Roche Diagnostics, China) was applied to lyse Huh-7 and HepG2 cells. Supernatants were incubated with antibody anti-AGO2 or IgG (EMD Millipore) along with protein A/G magnetic beads. Next, proteinase K and DNase (Beyotime, China) were used to remove proteins and DNA from the mixture. Subsequently, an RNeasy MinElute Cleanup Kit (Qiagen, China) was used to purify the RNA, and the purified RNA was analyzed through qRT-PCR.

### RNA pull-down experiment

Huh-7 and HepG2 cells were transfected with biotinylated miR-193a-5p. Then, the cells were rinsed with ice-cold phosphate buffer saline (PBS), harvested, and incubated on ice for 10 min in a lysis buffer (Sigma, USA). Afterward, the lysates were centrifuged at 12,000 *g* for 30 min at 4°C. The supernatant was incubated with M-280 streptavidin magnetic beads (Sigma). Then, the complex was eluted, the RNA in the complex was extracted, and qRT-PCR was executed to examine RNA levels.

### Western blot

Cells were lysed with RIPA buffer (Beyotime, China) and protein was harvested and quantified by a BCA kit (Beyotime). SDS-PAGE was employed to dissolve the protein extractions, then the protein was transferred to PVDF membranes (Sigma-Aldrich). Next, the membranes were blocked in 5% skim milk at room temperature for 1 h. The membranes were incubated with anti-MMP16 antibody (ab73877, 1:500, Abcam, UK), anti-MMP2 antibody (ab86607, 1:500, Abcam), anti-MMP9 antibody (ab38898, 1:500, Abcam), and anti-GAPDH antibody (ab8245, 1:3000, Abcam) for 12 h at 4°C. Next, the membranes were incubated with the secondary antibody (1:5000, Abcam) for 1 h at room temperature. An ECL Plus reagent (Life Technologies, USA) was added onto the membranes and the signals were detected using a chemiluminescence detection device (Bio-Rad).

### Statistical analysis

All the experiments were executed at least in triplicate. Statistical analysis was done with SPSS v22.0 (SPSS, Inc., USA). All data are reported as means±SD. To make the comparisons between two groups and among multiple groups, *t*-test and one-way ANOVA were used, respectively. The correlation was evaluated by Pearson's correlation coefficient. P<0.05 signified statistical significance.

## Results

### Circ_0001806 was up-modulated in HCC tissues and cell lines

**Figure 1:**
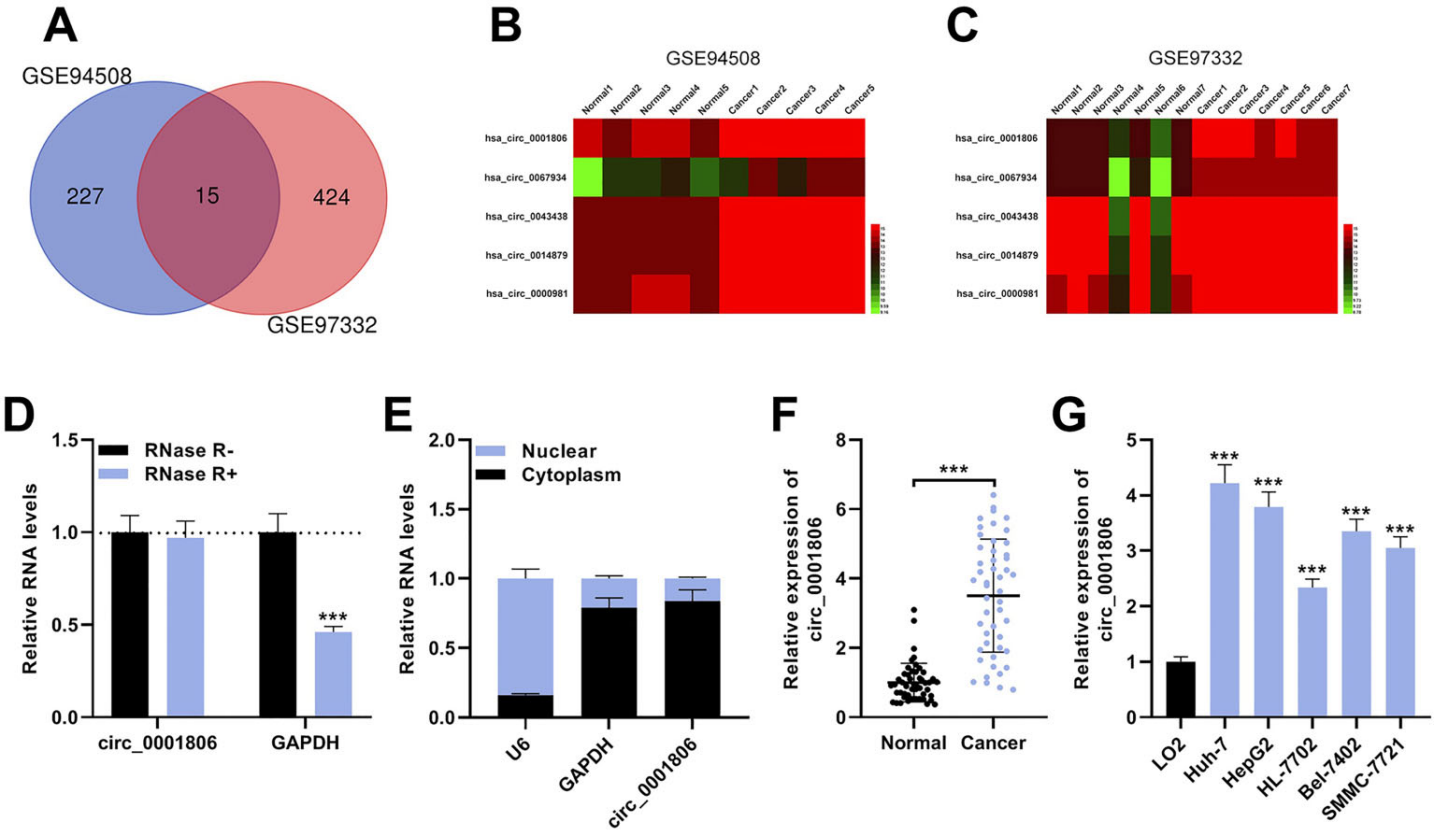
circ_0001806 is up-modulated in hepatocellular carcinoma (HCC). **A**, 15 circRNAs were differentially expressed in two microarray datasets (GSE94508, GSE97332) containing the circRNA expression profiles of HCC tissues and normal liver tissues. Heat maps showing the up-modulated circRNAs in HCC tissues compared to the matched non-tumor tissues GSE94508 (**B**) and GSE97332 (**C**). **D**, The levels of circ_0001806 were examined by qRT-PCR after treatment with RNase R. **E**, The subcellular distribution of circ_0001806 was analyzed by qRT-PCR after cellular RNA fractionation. GAPDH was used as the control for cytoplasm and U6 was used as the control for nucleus. **F**, The expression levels of circ_0001806 in 49 paired HCC tissues and matched adjacent normal tissues were examined by qRT-PCR. **G**, The expression levels of circ_0001806 in human HCC cell lines (Huh-7, HepG2, SMMC-7721, Bel-7402, and HL-7702) and the normal hepatic cell line (L02) were examined by qRT-PCR. Data are reported as means±SD. ***P<0.001 (ANOVA).

First of all, we analyzed the microarray data of GSE94508 and GSE97332 downloaded from the GEO database, with |log2(FC)|>1.5 and P<0.05 as criteria, and then obtained the differentially expressed circRNAs (DEcircRNAs) in HCC and in normal tissue samples with GEO2R tool (Figure 1A). There were 242 DEcircRNAs in GSE94508 and 439 DEcircRNAs in GSE97332. Fifteen circRNAs were differentially expressed in HCC samples in two datasets (Table 2). For instance, as shown in the heat map, 5 up-regulated circRNAs were displayed, including circ_0001806 (Figure 1B and C). Importantly, RNase R could degrade linear RNA GAPDH rather than circ_0001806, which indicated that circ_0001806 possessed a loop structure (Figure 1D). Furthermore, circ_0001806 was predominantly localized in the cytoplasm of L02 cells, suggesting it could probably function as a ceRNA (Figure 1E). Subsequently, qRT-PCR was adopted to examine circ_0001806 expression in 49 pairs of HCC tissues and non-tumor tissues. The data demonstrated that circ_0001806 expression in HCC tissues was higher than that in normal tissues adjacent to cancer (Figure 1F). We also measured circ_0001806 expression in HCC cell lines (Huh-7, HepG2, SMMC-7721, Bel-7402, and HL-7702) and it was significantly up-modulated compared with L02 cells (Figure 1G). These data revealed that circ_0001806 was overexpressed in HCC. We hypothesized that circ_0001806 might be a vital regulator in HCC progression.

**Table 2:** The intersected circRNAs form differentially expressed circRNAs in 2 gene sets.

Gene symbol	GSE94508		GSE97332	
	LogFC	P value	LogFC	P value
hsa_circ_0067934	1.831013	0.0223	2.6748404	0.00315
hsa_circ_0043438	1.554496	7.38E-09	2.0000682	0.0397
hsa_circ_0014879	1.548521	7.18E-09	1.5874528	0.0302
hsa_circ_0000981	1.541864	1.13E-08	1.6756029	0.0152
hsa_circ_0001806	1.539452	2.25E-09	2.3077778	0.000246
hsa_circ_0079480	1.524299	2.47E-06	-1.714286	4.57E-06
hsa_circ_0009581	-1.547747	0.000224	2.5098621	0.000521
hsa_circ_0084429	-1.566135	0.00311	1.7969988	0.000559
hsa_circ_0027774	-1.595124	0.000121	1.6627344	0.000397
hsa_circ_0056548	-1.759187	0.0000021	-1.66516	0.000954
hsa_circ_0007762	-1.951053	0.0000012	-1.553882	0.00238
hsa_circ_0078279	-2.060631	0.0000212	-1.501581	0.00155
hsa_circ_0059859	-2.064442	0.0000086	1.5601697	0.000159
hsa_circ_0055033	-2.843088	2.48E-06	2.6107158	0.0000107
hsa_circ_0004913	-6.093206	8.96E-13	-3.585576	0.0000169

Names in red indicate upregulated circRNAs in both gene sets; values in blue indicate the logFC value of circRNAs in both gene sets.

### Knockdown circ_0001806 repressed the growth and migration of HCC

**Figure 2:**
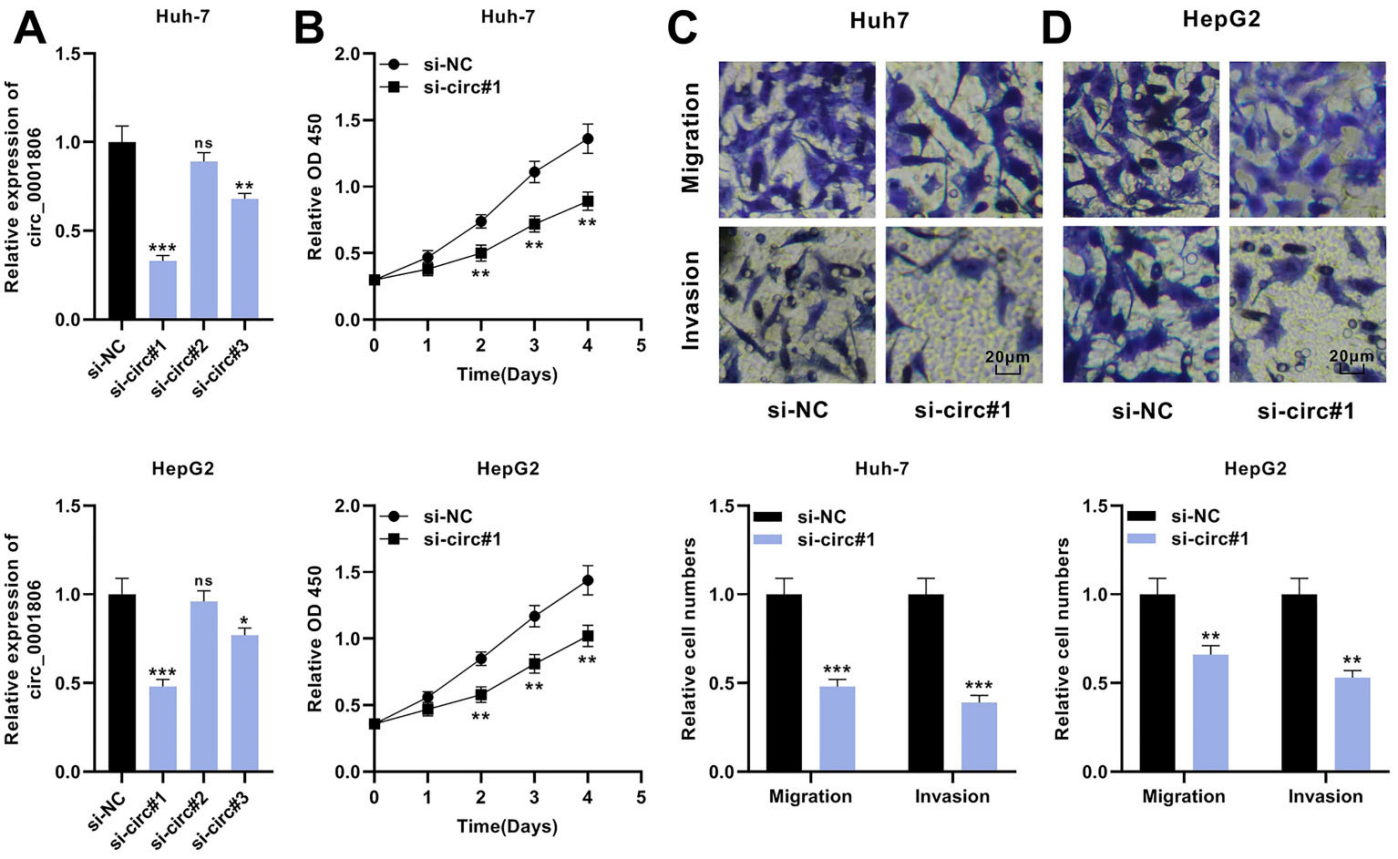
Knockdown of circ_0001806 inhibits multiplication, migration, and invasion of HCC cells. **A**, Three siRNAs targeting circ_0001806 (si-circ_0001806#1, si-circ_0001806#2, and si-circ_0001806#3) were transfected into Huh-7 and HepG2 cells and the expression level of circ_0001806 was detected by qRT-PCR. **B**, CCK-8 method was used to detect the multiplication of Huh-7 and HepG2 cells after transfection. **C** and **D**, Transwell experiment was employed to detect the migration and invasion of Huh-7 and HepG2 cells after transfection (scale bar 20 μm). Data are reported as means±SD. *P<0.05, **P<0.01, ***P<0.001; ns, not statistically significant (ANOVA and *t*-test).

Based on the above data, among the HCC cell lines, circ_0001806 expression was relatively higher in Huh-7 and HepG2 cells. Therefore, we transfected Huh-7 and HepG2 cells with 3 siRNAs targeting circ_0001806 (si-circ_0001806#1, si-circ_0001806#2, and si-circ_0001806#3), and si-circ_0001806#1 showed the best knockdown efficiency. Therefore, si-circ_0001806#1 was chosen for the follow-up experiments (Figure 2A). To probe the role of circ_0001806 in HCC, CCK-8 and Transwell methods were utilized to examine the multiplication, migration, and invasion of HCC cells. Relative to the control group, the proliferation of cells in circ_0001806 knockdown group was remarkably restrained (Figure 2B). Additionally, the migration and invasion of HCC cells in circ_0001806 knockdown group were also significantly repressed compared with those of the control group (Figure 2C and D). These data implied that knocking down circ_0001806 suppressed the proliferation, migration, and invasion of HCC cells, suggesting that circ_0001806 promoted HCC progression.

### Circ_0001806 targeted miR-193a-5p in HCC cells

**Figure 3:**
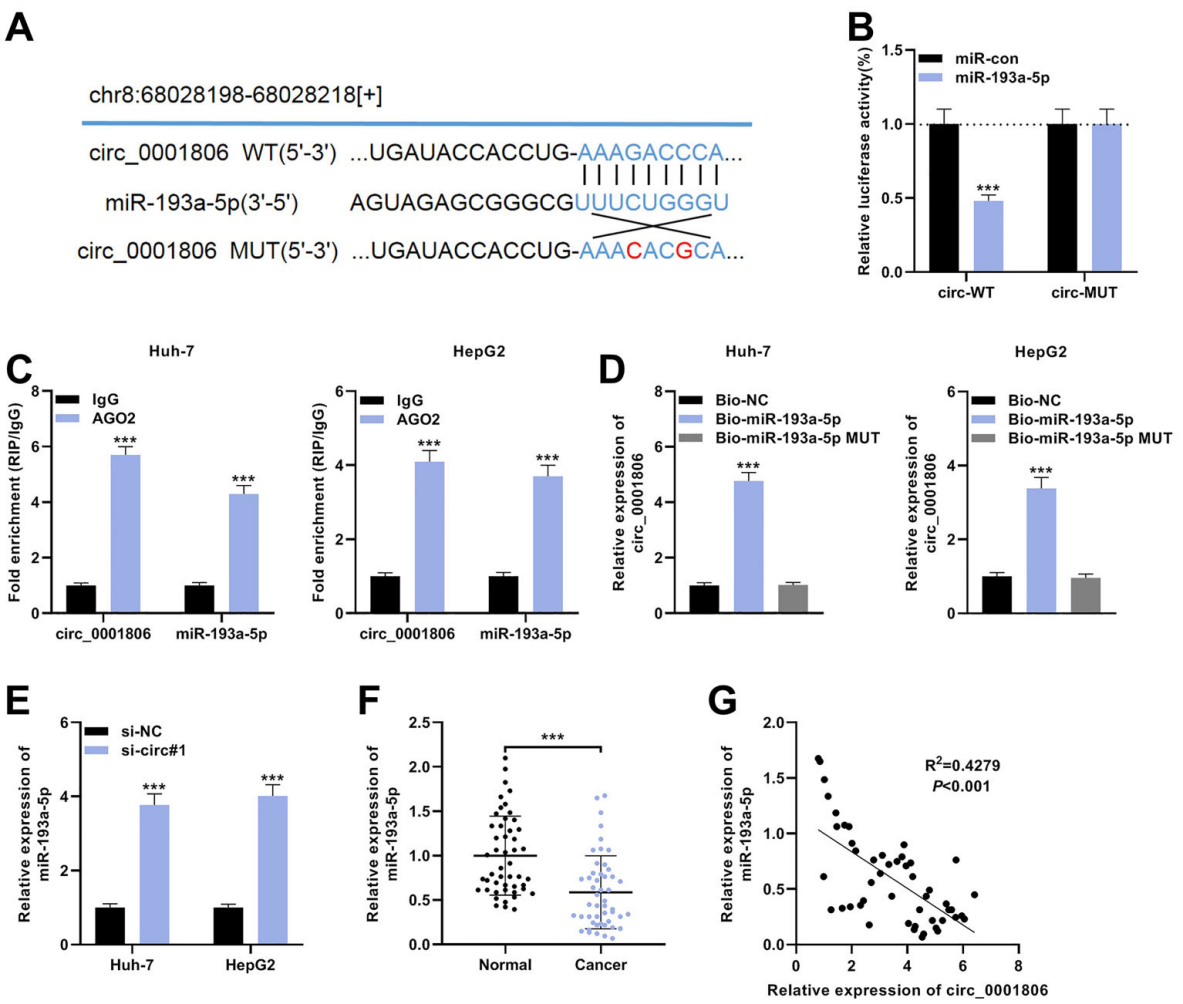
miR-193a-5p is the downstream target of circ_0001806. **A**, Bioinformatics analysis predicted that the sequence of miR-193a-5p matched the sequences of circ_0001806, and circ_0001806-WT (wild-type) and circ_0001806-MUT (mutant) luciferase reporter gene vectors were constructed. **B**, miR-193a-5p mimics (or miR control) were co-transfected with circ_0001806-WT (or circ_0001806-MUT) into HEK293T cells. After 48 h, the luciferase activity of the cells in each group was measured. **C**, The complex containing circ_0001806 and miR-193a-5p in Huh-7 and HepG2 cells was immunoprecipitated by anti-AGO2 using RIP assay. **D**, RNA pull-down assay was carried out to verify the interactions between circ_0001806 and miR-193a-5p. **E**, qRT-PCR was used to detect the expression of miR-193a-5p in Huh-7 and HepG2 cells after circ_0001806 knockdown. **F**, qRT-PCR was used to examine the expression levels of miR-193a-5p in 49 paired hepatocellular carcinoma (HCC) tissues and matched adjacent normal tissues. **G**, Pearson correlation analysis was utilized to evaluate the correlation between the expressions of circ_0001806 and miR-193a-5p in HCC tissues. Data are reported as means±SD. ***P<0.001 (*t*-test).

To determine the downstream mechanism of circ_0001806 in HCC progression, the starBase database (http://starbase.sysu.edu.cn/) was searched, and it was predicted that miR-193a-5p was a potential downstream target of circ_0001806 (Figure 3A, Supplementary Figure S1). To verify the targeting relationship between circ_0001806 and miR-193a-5p, we co-transfected circ_0001806 wild-type plasmid (circ_0001806-WT) or mutant plasmid (circ_0001806-MUT) with miR-193a-5p mimic or control (miR-control) into HEK293T cells, respectively. The luciferase reporter gene assay revealed that, compared with the control group, miR-193a-5p mimics significantly repressed the luciferase activity of cells transfected with circ_0001806-WT plasmid, but exerted no significant effect on the luciferase activity of cells transfected with circ_0001806-MUT plasmid (Figure 3B). RIP and pull-down data revealed that circ_0001806 could bind with miR-193a-5p in both Huh-7 and HepG2 cells (Figure 3C and D). Moreover, after knocking down circ_0001806, miR-193a-5p expression in Huh-7 and HepG2 cells was significantly augmented (Figure 3E). In addition, data revealed that miR-193a-5p was significantly under-expressed in HCC tissues compared with normal tissues adjacent to cancer (Figure 3F). Pearson's correlation analysis showed that miR-193a-5p expression was negatively correlated with circ_0001806 expression (R^2^=0.4279, P<0.001) (Figure 3G). These data suggested that circ_0001806 adsorbed miR-193a-5p and negatively modulated its expression in HCC cells.

### MMP16 was one of the direct targets of miR-193a-5p in HCC

**Figure 4:**
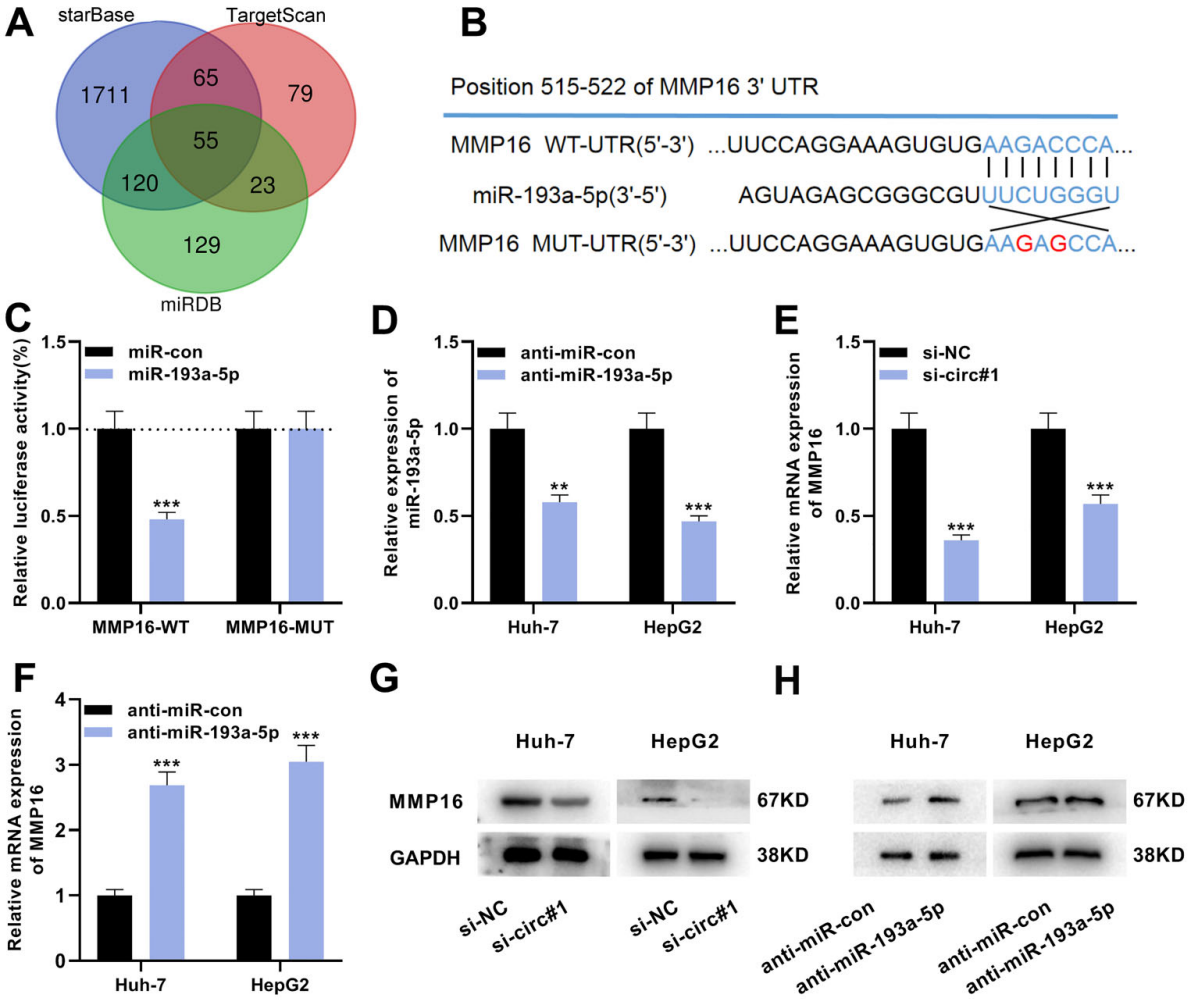
MMP16 is the direct target of miR-193a-5p. **A**, Bioinformatics analysis was performed with starBase, TargetScan, and miRDB to predict the candidate mRNAs, which could be targeted by miR-193a-5p. **B**, Bioinformatics analysis predicted that the sequence of MMP16 3UTR matched the sequences of miR-193a-5p, and MMP16-WT (wild-type) and MMP16-MUT (mutant) luciferase reporter gene vectors were constructed. **C**, The miR-193a-5p mimics (or miR control) were co-transfected with MMP16-WT (or MMP16-MUT) into HEK293T cells, and the luciferase activity of the cells in each group was measured. **D**, Huh-7 and HepG2 cells were transfected with anti-miR-193a-5p and negative control (NC), and the expression level of miR-193a-5p was detected by qRT-PCR. **E**, qRT-PCR was used to detect the expression of MMP16 mRNA in Huh-7 and HepG2 cells after circ_0001806 knockdown. **F**, qRT-PCR was used to detect the expression of MMP16 mRNA in Huh-7 and HepG2 cells transfected with anti-miR-193a-5p. **G**, Western blot was used to detect the expression of MMP16 protein in Huh-7 and HepG2 cells after circ_0001806 knockdown (**G**) and in Huh-7 and HepG2 cells transfected with anti-miR-193a-5p (**H**). Data are reported as means±SD. **P<0.01, ***P<0.001 (*t*-test).

Next, bioinformatics analysis was conducted with starBase, TargetScan (www.targetscan.org), and miRDB (www.mirdb.org) to identify candidate mRNAs targeted by miR-193a-5p, and analysis revealed that MMP16 3′UTR and miR-193a-5p had complementary binding sites (Figure 4A and B). GEPIA database (http://gepia.cancer-pku.cn/) indicated that the overall survival of patients with high MMP16 expression was significantly shorter than that of patients with low MMP16 expression (Supplementary Figure S2). The data of the luciferase reporter gene experiment showed that the luciferase activity of the cells co-transfected with the luciferase reporter plasmid MMP16-WT and miR-193a-5p mimic was significantly diminished, while the luciferase activity of the cells transfected with MMP16-MUT was unchanged (Figure 4C). Furthermore, anti-miR-193a-5p was transfected into Huh-7 and HepG2 cell lines to construct an miR-193a-5p under-expression model (Figure 4D). qRT-PCR showed that after knocking down circ_0001806, MMP16 mRNA expression in Huh-7 and HepG2 cells was significantly decreased, while after miR-193a-5p expression was inhibited, and MMP16 mRNA expression in Huh-7 and HepG2 cells was remarkably augmented (Figure 4E and F). Additionally, western blot data were consistent with the results of qRT-PCR (Figure 4G and H). The findings indicated that MMP16 was a direct target of miR-193a-5p, and circ_0001806 could positively regulate MMP16 expression in HCC cells.

### Circ_0001806 enhanced the multiplication, migration, and invasion of HCC cells through the miR-193a-5p/MMP16 axis

**Figure 5:**
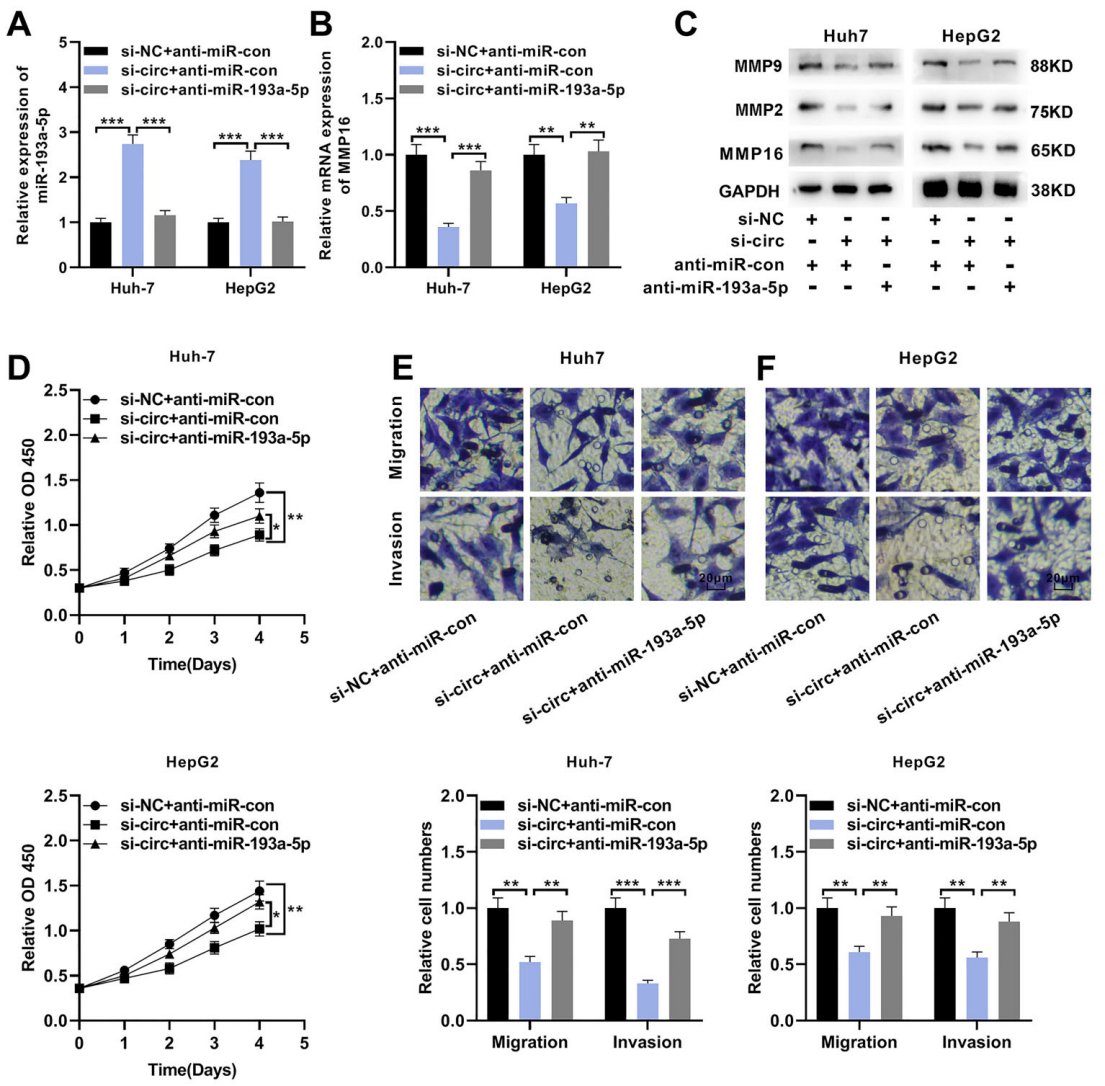
Circ_0001806 enhances the multiplication and invasion of hepatocellular carcinoma (HCC) cells through miR-193a-5p/MMP16 axis. Huh-7 and HepG2 cells were transfected with si-NC + anti-miR-Con, si-circ_0001806#1 + anti-miR-Con, or si-circ_0001806#1 + anti-miR-193a-5p. **A**, qRT-PCR was used to detect the expression of miR-193a-5p in Huh-7 and HepG2 cells. **B**, qRT-PCR was used to detect the expression of MMP16 mRNA in Huh-7 and HepG2 cells. **C**, Western blot was used to detect the expression of MMP16, MMP2, and MMP9 protein in Huh-7 and HepG2 cells. **D**, CCK-8 method was used to detect the multiplication of Huh-7 and HepG2 cells after transfection. The transwell experiment was employed to detect the migration and invasion of Huh-7 cells (**E**) and HepG2 cells (**F**) after transfection (scale bar 20 μm). Data are reported as means±SD. *P<0.05, **P<0.01, and ***P<0.001 (ANOVA).

To assess whether circ_0001806 affected HCC progression by modulating the miR-193a-5p/MMP16 axis, compensation experiments were performed. Anti-miR-193a-5p or negative control (anti-miR-control) was co-transfected into Huh-7 and HepG2 cell lines transfected with si-circ_0001806#1 (Figure 5A). Inhibition of miR-193a-5p partly counteracted the inhibitory effect of circ_0001806 knockdown on MMP16 expression in Huh-7 and HepG2 cells (Figure 5B and C). Western blot results also showed that circ_0001806 knockdown could inhibit the expression of MMP2 and MMP9, but inhibition of miR-193a-5p partly counteracted this effect (Figure 5C). CCK-8 experiment data revealed that knocking down circ_0001806 repressed the multiplication of Huh-7 and HepG2 cells, while down-modulating miR-193a-5p partly counteracted this effect (Figure 5D). Additionally, the data of the Transwell experiment suggested that knocking down circ_0001806 impeded the migration and invasion of Huh-7 and HepG2 cells, while this effect could be reversed by the inhibition of miR-193a-5p (Figure 5E and F). Collectively, it was concluded that circ_0001806 enhanced the multiplication, migration, and invasion of HCC cells via the miR-193a-5p/MMP16 axis.

## Discussion

HCC is one of the most deadly malignancies (12,13). Clarifying the mechanism of HCC progression is crucial to develop novel biomarkers and therapy targets for this disease (14,15). Several circRNAs are known to play roles in the tumorigenesis and disease progression of HCC (16-19). For instance, circ-ADD3 is under-expressed in HCC and it represses the metastasis of HCC by modulating EZH2 (17). Circ_0000517 is up-modulated in HCC and it contributes to HCC progression by modulating MAPK and Ras pathways (18). The expression of circ-PRKCI is associated with poor prognosis in HCC patients, and circ-PRKCI could inhibit cell apoptosis and promote cell invasion (19). Furthermore, knocking down circ_0000092 restrains HCC cell multiplication, migration, invasion, and angiogenesis by down-modulating HN1 (20). In this work, we focused on the expression characteristics, biological function, and underlying mechanism of circ_0001806 in HCC. Circ_0001806 is reported to be remarkably up-modulated in colorectal cancer tissues and it is linked to TNM staging, depth of invasion, lymphatic metastasis, and distant metastasis (21). In this work, we demonstrated that circ_0001806 was also overexpressed in HCC tissues and cell lines. Additionally, circ_0001806 knockdown repressed the multiplication, migration and invasion of HCC cells. These findings indicated that circ_0001806 facilitated HCC progression.

Some circRNAs work as ceRNAs to regulate miRNAs, and this mechanism is common in cancer biology (22,23). For instance, circ_0001649 can function as ceRNA to absorb miR-127-5p, miR-612, and miR-4688 and activate SHPRH to repress HCC progression (24). Down-regulating circ_0005986 enhances cell multiplication by increasing miR-129-5p and decreasing Notch1 expression to facilitate cell cycle progression of HCC cells (25). Reportedly, miR-193a-5p is under-expressed in HCC, and miR-193a-5p suppresses the malignancy of HCC cells (26,27). This suggests that miR-193a-5p is a tumor suppressor in HCC. In this work, circ_0001806 was found to target miR-193a-5p and repress its expression level in HCC cells, which suggested that the abnormal high expression of circ_0001806 contributed to the under-expression of miR-193a-5p in HCC. Also, we demonstrated that inhibiting miR-193a-5p partially counteracted the effects of circ_0001806 knockdown on HCC cells, and this finding indicated that circ_0001806 partly exerts its biological function via regulating miR-193a-5p in HCC cells.

MMP16 is a membrane-type metalloprotease that facilitates cancer progression via multiple mechanisms (28,29). MMP16 can degrade the components of extracellular matrix and facilitate the migration and invasion of cancer cells (29). Reportedly, MMP16 is abnormally expressed in diverse cancers such as melanoma, lung cancer, endometrial cancer, prostate cancer, and others (29-33). For instance, MMP16 is overexpressed in gastric cancer and is linked to unfavorable prognosis (33). Moreover, MMP16 is validated as the target gene of diverse miRNAs, such as miR-132, miR-328-3p, miR-33b, and miR-26a (34-37). These studies suggest that miRNAs are crucial regulators of MMP16 expression. In HCC, it is reported that MMP16 overexpression enhances HCC metastasis by modulating epithelial-mesenchymal transition process (38). In this work, we demonstrated that MMP16 was identified as a target gene of miR-193a-5p, and circ_0001806 positively modulated MMP16 expression by repressing miR-193a-5p. These data support that the ceRNA network consisting of circ_0001806, miR-193a-5p, and MMP16 is a novel mechanism of HCC progression. MMP9 and MMP2 are also crucial regulators of migration and invasion in HCC (39,40). Interestingly, we also demonstrated that circ_0001806 / miR-193a-5p axis not only regulated the expression of MMP16 in HCC cells, but also regulated the expression of MMP2 and MMP9. Our data suggested that circ_0001806 and miR-193a-5p are important regulators of matrix metalloprotease members, but the detailed mechanisms remain to be deciphered in following studies.

In summary, our research showed that circ_0001806 was up-modulated in HCC tissues and cells. Circ_0001806 knockdown repressed the multiplication and metastatic potential of HCC cells. Mechanistically, circ_0001806 participated in HCC progression through the miR-193a-5p/MMP16 axis. Our data suggested that circ_0001806 is a promising potential biomarker and therapy target for HCC.

## Supplementary Material

Click here to view [pdf].
